# Durability Performance of Geopolymer Concrete: A Review

**DOI:** 10.3390/polym14050868

**Published:** 2022-02-23

**Authors:** Leong Sing Wong

**Affiliations:** College of Graduate Studies, Universiti Tenaga Nasional, Jalan IKRAM-UNITEN, Kajang 43000, Selangor, Malaysia; wongls@uniten.edu.my

**Keywords:** geopolymer concrete, alkaline activator, aluminosilicate sources, durability, compressive strength, chloride ion penetrability, acid attack, abrasion

## Abstract

Geopolymer concrete is produced from the geopolymerization process, in which molecules known as oligomers integrate to form geopolymer networks with covalent bonding. Its production expends less thermal energy and results in a smaller carbon footprint compared to Ordinary Portland Cement (OPC) concrete. It requires only an alkaline activator to catalyze its aluminosilicate sources such as metakaolin and fly ash, to yield geopolymer binder for the geopolymerization to take place. Because of its eco-friendly technology and practical application, current research interest is mainly concentrated on the endurance of geopolymer concrete to resist heat and chemical aggressions. As such, it is pertinent for this review article to provide critical insight into the recent progress in research on the durability of geopolymer concrete. One significant outcome of the review is that the admixture of geopolymer concrete could be blended with additives such as micro-silica and fibers such as polypropylene fibers, to enhance its durability. The review on the durability aspects of geopolymer concrete showed that it had high compressive strength at an optimal elevated temperature, low to medium chloride ion penetrability, and high resistance to acid attack and abrasion. This makes geopolymer concrete a viable candidate to replace OPC concrete in the construction industry.

## 1. Introduction

Approximately 5 to 7% of global carbon dioxide (CO_2_) emissions can be attributed to Ordinary Portland Cement (OPC), which has traditionally been used as the primary binder in concrete [1]. A typical mixture of concrete has a cement content ranging from 15 to 20%. In fact, cement manufacturing consumes a lot of energy, and a high temperature of 1450 °C is required for heating its raw materials in a cement kiln. OPC production demands high levels of energy consumption, mainly due to the high temperatures needed for clinker production but also due to the milling of the raw materials and the clinker [2]. This causes a massive emission of CO_2_ to the environment. Until now, cement has been widely used as a binding agent in the construction industry because of its capability to bind aggregate in the presence of water to form cement-based materials of various sizes and shapes [3]. Such a consequence accelerates global warming and promotes climate change. Phenomenal global warming and a consequent rise in seawater level are causing earth land to shrink [4]. This may affect the quality of life of the world’s population [5].

Geopolymerization is viewed as an attractive process for producing eco-friendly concrete. Geopolymers are important alternative materials for use in support of recycling and sustainability [6]. The geopolymerization of aluminosilicates constitutes a radical change in construction materials chemistry and synthesis pathways compared with the calcium silicate hydrate chemistry which underpins Portland cement [7]. The binder of geopolymer concrete is geopolymer cement, which is formulated from alkaline activated aluminosilicates of natural clays or by-products from the industry. Alkaline activation requires essentially two components: a precursor and an alkaline activator [8]. In regard to that, aluminosilicates such as rice husk ash, silica fume, fly ash, and metakaolin are conventionally applied as precursors of geopolymer cement. Unlike OPC, less energy utilization of raw materials is required for geopolymer cement production. The source of energy consumption, which is required to produce geopolymer concrete, could be due to sodium hydroxide production, the activating solution preparation, and external heat for curing if it is presented [9]. In addition, sodium silicate requires less thermal energy in its manufacturing. As such, it produces less carbon emission than OPC. The usage of thermal energy for the production of sodium silicate requires a temperature of 1100 °C, which is 350 °C lower than that needed for the making of OPC. Geopolymer concrete is considered as a possible alternative to OPC concrete [10]. Unlike normal concrete, the matrix formation and strength development of geopolymer concrete do not need calcium silicate hydrate (C-S-H) gel to be form [11]. The cementation phenomenon of geopolymer concrete is caused by the polycondensation of aluminosilicate. Marked structural, mechanical, and physical differences have been identified in activated binders using precursors from different sources, as a consequence of the chemical and physical differences between slag and fly ash precursors and the influences of different activator concentrations and chemistries, and the microstructures of alkali-activated slag and of fly ash geopolymers have been extensively studied in systems based on sole precursors [12]. Fly ash has been used for producing geopolymeric cement with mechanical strength up to around 60 MPa [13]. Provis et al. [14] have identified ten types of precursor that can be applied to develop geopolymerized materials. They are silico-manganese slag, mineral processing tailings, catalyst residues, coal bottom ash, rice husk ash, palm oil fuel ash, waste glass, waste ceramic, incineration products of sludges, and natural minerals.

Based on geopolymerization, the durability of geopolymer concrete is attributable to the development of sodium aluminosilicate hydrate (N-A-S-H) gel. Lodeiro et al. [15] provided a detailed description on the geopolymerization mechanism that involved three sequential stages, namely, destruction-coagulation, coagulation-condensation, and condensation-crystallization. The process of geopolymerization starts with the degradation of the aluminosilicate structure by hydroxide ions from an alkaline activator. Such process is governed by the amount of dissolution of silicate and aluminate species. Interactions between the small dissolved species, and involving any silicate initially supplied by the activating solution, lead to the formation of aluminosilicate oligomers [16]. The dissolution of the species continues until the dissolved aluminate is concentrated enough to weaken the dissolved silicate, which eventually causes precipitation of N-A-S-H gel. Under such conditions, the development of N-A-S-H gel progresses with time to form solid N-A-S-H crystals, which eventually contribute to geopolymerized bonding. The duration required for the N-A-S-H crystallization is dependent on the design of the geopolymer admixture and the temperature of curing.

It has been shown that geopolymerization can transform a wide range of waste aluminosilicate materials into building and mining materials with excellent chemical and physical properties, such as fire and acid resistance [17]. Self-compacting geopolymer concrete with zero cement and zero superplasticizers cured under ambient conditions was tested to have 40 MPa compressive strength after 28 days of curing, which is comparable to that of M40 grade conventional concrete [18]. The strength of geopolymer concrete was reported to increase with an increase of curing time and a rise in temperature, as reported in several studies [19,20,21]. However, Zhang et al. [22] clarified that the strength of geopolymer concrete deteriorated after exposure to an optimal curing temperature. The dehydration of the geopolymer was identified by Zhang et al. [22] as a reason for the strength loss of the geopolymer concrete after exposure to a temperature of higher than 600 °C. Chen et al. [23] reported that durability of geopolymer concrete is better than OPC concrete, and calcium content has a great effect on the durability mechanism. Any concrete structure should be durable and able to fend off the processes of debilitation to which it is anticipated to be exposed [24].

**Figure 1:**
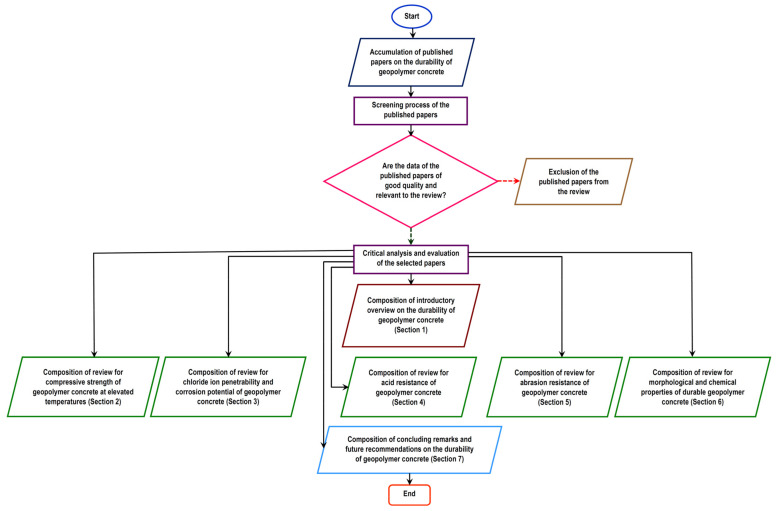
Flow chart of the review of the durability of geopolymer concrete.

Although Chen et al. [23] have reviewed the durability of geopolymer concrete exposed to an aggressive environment, comprehensive literature with regard to the source of aluminosilicate, the type of reinforcing raw material, and the mix design that can optimize long-term robustness of the construction material are lacking. Saranya et al. [25] found that a mixture of ground-granulated blast furnace slag and dolomite had a profound effect on improving the load carrying capacity of geopolymer concrete beam-column joints under monotonic loading. The geopolymer concrete beam-column joints were tested to have better ductility, greater energy absorptivity, and higher toughness with the inclusion of steel fibers. Since steel-fiber-reinforced alkali-activated geopolymer concrete can achieve higher mechanical performance and produce less carbon emission as compared to the conventional concrete, it is considered to be a potential construction material solution for the buried tunnel subjected to gas explosion threatening [26]. In another study, Alrshoudi et al. [27] added glass and carbon-fiber-reinforced polymers to high-strength geopolymer concrete to enhance its compressive strength and ductility. Fiber-reinforced concrete also demonstrates other fair qualities of durability in terms of drying shrinkage, chloride ion penetration, water permeability, abrasion resistance, and impact resistance [28]. In the study of Chithambar Ganesh et al. [29], plastic wastes in the form of polyethylene terephthalate (PET) bottles were ground into powder and partially incorporated in geopolymer concrete. The compressive strength and split tensile strength of the geopolymer concrete were reported to increase by 5.8 and 24%, respectively, with a 10% usage of the plastic powder as a partial replacement of sand. Geopolymers blended recycled concrete (OPC, fly ash, rice husk ash, river sand, Cupola furnace slag, and crushed granite) was discovered by Alabi and Mahachi [30] to have better penetration resistance to chloride ion than other concrete mixes in the study and are therefore more durable. With low chloride ion penetrability, it could be affirmed that the geopolymer-blended recycled concrete offered good protection to steel reinforcement against corrosion. It is notable that there are a number of published research papers [31,32,33,34,35,36,37,38,39,40,41,42,43,44,45,46,47] on the mechanical properties of geopolymer concrete without revealing the findings on its durability characteristics. Guided by these developments, it is the aim of this article to review and evaluate the durability aspects of geopolymer concrete. Any durable geopolymer concrete should be capable to resist weathering action, chemical degradation, and abrasion. This review provides an important insight into the ability of geopolymer concrete to withstand deterioration over time and harsh conditions. Figure 1 illustrates the flow chart of the review on durability of geopolymer concrete. Essentially, the review concentrated on five sectional topics relevant to the durability of geopolymer concrete. These sectional topics are compressive strength at elevated temperatures, chloride ion penetrability and corrosion potential, acid resistance, abrasion resistance, and the morphological and chemical properties of geopolymer concrete.

## 2. Compressive Strength of Geopolymer Concrete at Elevated Temperatures

Residual compressive strength of a material is referred to as the maximum compressive stress that the damaged material can sustain under a loading application. The resistance of geopolymer concrete to crack propagation influences its residual compressive strength. The fracture toughness and residual compression resistance of geopolymer concrete tend to decrease when it is exposed to a very high temperature. High temperature, i.e., heat exposure, is one of the most important parameters that affects the surface characteristics, surface outlook, shape, and color of concrete [48]. Therefore, residual compressive strength is an important durability characteristic of geopolymer concrete. The residual compression resistance of geopolymer concrete at elevated temperatures were investigated in numerous studies.

**Figure 2:**
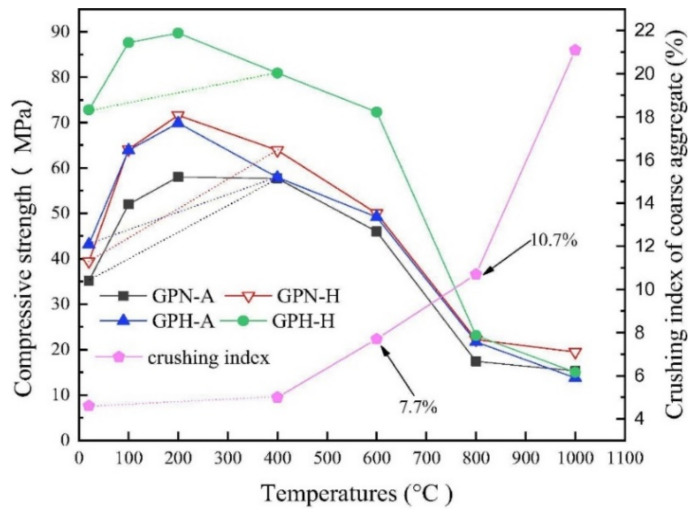
Compressive strength of geopolymer concrete specimens and crushing index of coarse aggregate at elevated temperatures (note: GPN-A, ambient-cured normal strength geopolymer concrete; GPN-H, heat-cured normal strength geopolymer concrete; GPH-A, ambient-cured high-strength geopolymer concrete; and GPH-H, heat-cured high-strength geopolymer concrete). Reproduced with permission from [22], [Construction and Building Materials]; published by [Elsevier], [2020].

**Figure 3:**
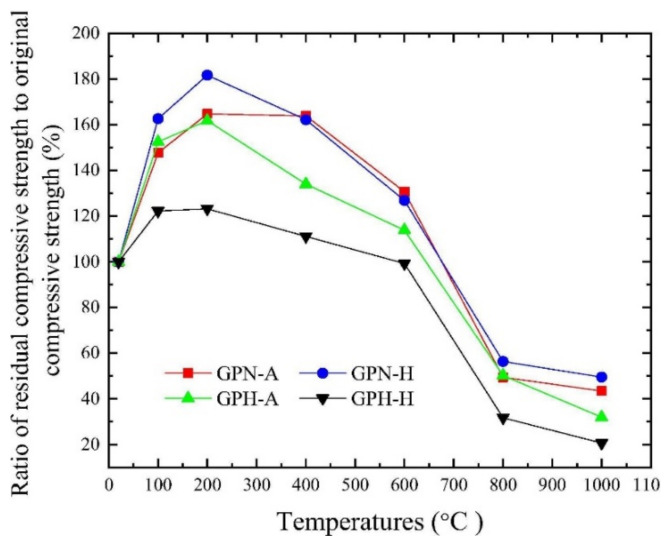
The ratios of residual to original compressive strength of geopolymer concrete specimens at elevated temperatures based on Figure 2, Reproduced with permission from [22], [Construction and Building Materials]; published by [Elsevier], [2020].

Zhang et al. [22] evaluated the residual compression resistance of four types of geopolymer concrete, which were exposed to high temperatures ranging from 20 to 1000 °C for 2 h. The heating rate applied to the geopolymer concrete was 5 °C min^−1^. The four types of concrete are ambient-cured normal-strength geopolymer concrete (GPN-A), heat-cured normal strength geopolymer concrete (GPN-H), ambient-cured high-strength geopolymer concrete (GPH-A), and heat-cured high-strength geopolymer concrete (GPH-H). The ambient and heat curing temperatures are 25 and 80 °C, respectively. The respective sodium-silicate-to-sodium-hydroxide ratios for the normal and high-strength geopolymer concrete are 2.5 and 3.0. Figure 2 shows the compressive strength and crushing index of coarse aggregate of the geopolymer concrete specimens at elevated temperatures. Between 20 and 400 °C, the compressive strength values of all the geopolymer concrete specimens were found to be above their original compressive strength values. This could be attributed to the low crushing index of coarse aggregate within the temperature range depicted in Figure 2. The findings reflected the ability of coarse aggregate in the geopolymer concrete specimens to withstand thermal stress up to 400 °C. As expected, the GPH-H had the highest compressive strength of 89.9 MPa at 200 °C. At the same temperature, its ratio of elevated temperature compressive strength to original compressive strength was found to be 123.2%, which is the lowest among all the geopolymer concrete specimens tested (Figure 3). The study revealed that the maximum temperature that all the geopolymer concrete specimens can endure before strength loss is 600 °C. Hence, Zhang et al. [22] clarified that the threshold crushing index of coarse aggregate of the geopolymer concrete specimens is 7.7% at 600 °C. Beyond 600 °C, the geopolymer concrete specimens experienced cracking and spalling due to thermal degradation of the coarse aggregate, which caused their compressive strength to decrease even further. It was found that the percentage residual strength of GPH-A was higher than that of GPH-H at all the temperatures, indicating that ambient-cured geopolymer concrete performed better compressive strength enhancement and lower strength deterioration than the heat-cured ones [22].

**Figure 4:**
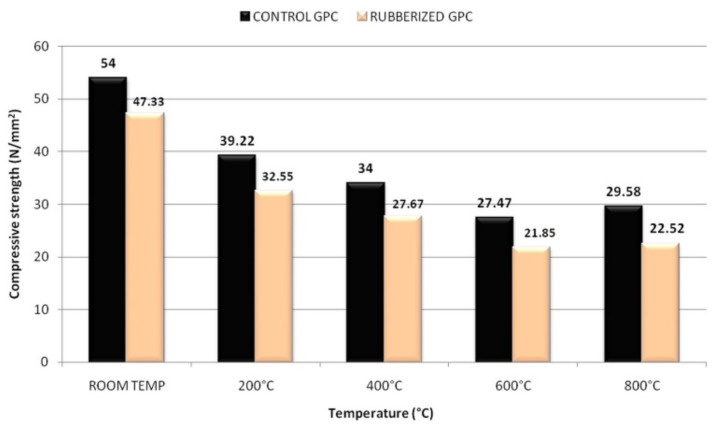
Compressive strength of control and rubberized geopolymer concrete specimens after exposure to elevated temperature (note: GPC, geopolymer concrete). Reproduced with permission from [49], [Journal of Building Engineering]; published by [Elsevier], [2018].

In another development, Luhar et al. [49] investigated the compressive strength of fly-ash-based control and rubberized geopolymer concrete specimens (CONTROL GPC and RUBBERIZED GPC) at room temperature, and at 200, 400, 600, and 800 °C. Starting from room temperature, each geopolymer concrete specimen was heated at a rate of 4.4 °C min^−1^ until an elevated temperature was achieved, and after that the elevated temperature was maintained for a duration of 2 h. The trend of the compressive strength development of the geopolymer concrete specimens is depicted in Figure 4. It is observed in Figure 4 that for all the temperatures, the compressive strength reductions of the RUBBERIZED GPC were slightly lower than the CONTROL GPC. The percentage reductions of compressive strength for the RUBBERIZED GPC were noticed to be 31.23 and 52.43% at the respective temperatures of 200 and 800 °C. On the other hand, the percentage values of the strength reduction for the CONTROL GPC were realized to be 27.38 and 45.22% at 200 and 800 °C, respectively. This was attributed to the soft and light-weight nature of the rubber tire fibers, which could induce cracks of the geopolymer concrete specimens at the initial compression stage. The tendency of the rubber tire fibers to entrap air and form pores in the RUBBERIZED GPC resulted in their higher strength reductions at elevated temperatures compared to the CONTROL GPC.

**Figure 5:**
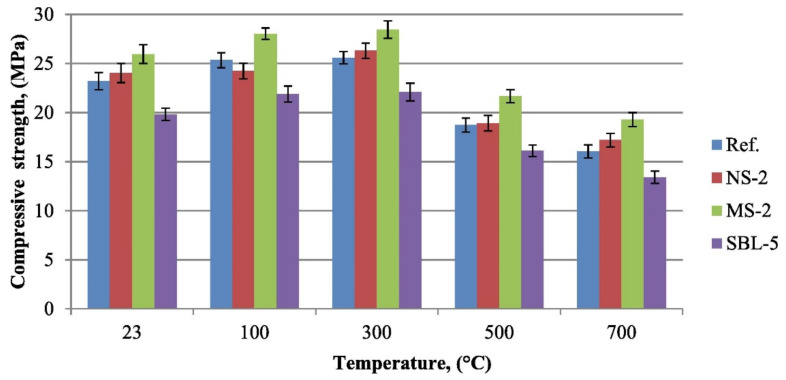
Compressive strength of geopolymer concrete samples after elevated temperatures (note: Ref, control geopolymer concrete; NS-2, geopolymer concrete with 2% nano-silica additive; MS-2, geopolymer concrete with 2% micro-silica additive; and SBL-5, geopolymer concrete with 5% styrene-butadiene latex). Reproduced with permission from [50], [Construction and Building Materials]; published by [Elsevier], [2021].

Kantarci et al. [50] studied the compressive strength of volcanic-tuff-based geopolymer concrete using nano-silica, micro-silica, and styrene-butadiene latex at elevated temperatures. Figure 5 indicates the compression resistance of the improved geopolymer concrete samples compared to the control ones at the temperatures of 23, 100, 300, 500, and 700 °C. Each geopolymer concrete sample was heated at an elevated temperature for 1 h. The compressive strength of all geopolymer concrete specimens increased as the temperature rose from 23 to 300 °C. The increase in the compressive strength may be due to promotion of polycondensation between tetrahedral aluminosilicate gels [50]. The process involved the condensation reactions that caused geomonomers to combine and form the geopolymers in the concrete, and this was accompanied by the expulsion of water during the process of heating. At a temperature of 300 °C, the geopolymer concrete sample with 2% micro-silica additive had the highest compressive strength of 28.45 MPa compared to all the other samples of geopolymer concrete under study. At the same temperature, the geopolymer concrete samples with 2% nano-silica additive, without additive, and with 5% styrene-butadiene latex were tested to have compressive strength of 26.30, 25.60, and 22.09 MPa, respectively. This proved that the type of additive in the geopolymer concrete sample played an important role in enhancing the polycondensation reactivity that resulted in its high compressive strength at the elevated temperature. When the geopolymer concrete samples were exposed to a temperature higher than 300 °C, their compressive strength declined due to the formation of micro-cracks in them as a result of their degradation caused by extreme heat.

**Figure 6:**
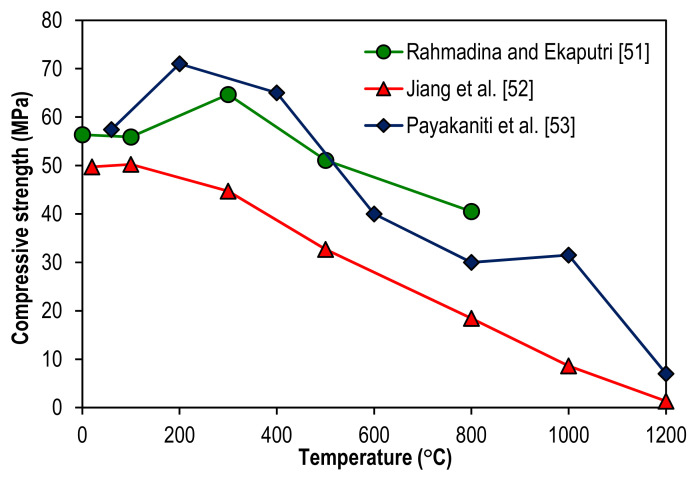
Compressive strength of geopolymer specimens at elevated temperatures from the published works of Rahmadina and Ekaputri [51], Jiang et al. [52], and Payakaniti et al. [53].

Based on three literature studies, the impact of geopolymerization methods on the compressive strength of geopolymer specimens at elevated temperatures can be further examined from Figure 6. The descriptions for the geopolymerization methods and chemical compositions of the geopolymer specimens are summarized in Table 1. After investigating the application of Class F fly ash as a precursor for geopolymerization, Rahmadina and Ekaputri [51] found that compressive strength of geopolymer specimen peaked at 71.00 MPa after exposure to 200 °C. It then decreased by 8.45, 43.66, 57.75, 55.63, and 90.14% when the temperatures were elevated at 400, 600, 800, 1000, and 1200 °C respectively. Using the same precursor for geopolymerization but with a higher molar concentration of sodium hydroxide and higher water content, Jiang et al. [52] discovered that compressive strength of geopolymer specimen reached its peak at 64.70 MPa after exposure to 300 °C. The strength was later reduced by 21.02 and 37.40% at the respective temperature of 500 and 800 °C. This indicates that a careful design of the geopolymer specimen admixture is required to maximize its compressive strength and optimize its elevated temperature. In the study of Payakaniti et al. [53], the method of alkaline activation by sodium hydroxide and sodium silicate solutions with high calcium (Class C) lignite fly ash as a precursor was applied for the making of geopolymer specimens. The highest compressive strength of the geopolymer specimen under the study is 50.24 MPa after exposure to 100 °C. The strength declined consistently by 11.01, 34.95, 63.28, 82.84, and 97.37% at 300, 500, 800, 1000, and 1200 °C respectively. This reflects that different types of precursors induced different levels of geopolymerization reactivity. There was a threshold temperature for the geopolymerization reaction to peak and failure was noticed in the geopolymer specimens after exposure beyond the threshold temperature due to thermal pressure. As a result of the thermal instability due to high temperatures, cracks were induced in the geopolymer specimens. This led to the decline in their compressive strength. A similar trend of compressive strength development of geopolymer specimens at elevated temperatures was also reported by Liu et al. [54]. In the study of Liu et al. [54], it was clarified that metakaolin-based geopolymer specimens were covered with ductile crack markedly after high-temperature exposure at 300 °C, and the crack further developed into fork crack after 500 and 700 °C exposure.

In summary, it can be deduced from Section 2 that the compressive strength of geopolymer concrete at an elevated temperature could be optimized using additives such as fly ash, nano-silica and micro-silica. Inclusion of rubber tire fibers could slightly decrease the compressive strength of geopolymer concrete at an elevated temperature due to its role at causing concrete cracks upon initial loading. The negative residual strength impact of the rubber tire fibers on the geopolymer concrete was due to the fibers’ capability to capture air and form pores during compression test. As such, it is interesting to explore other additive such as ultra-fine calcium carbonate on the compressive strength development of geopolymer concrete for a range of high temperatures. Geopolymer composites, if designed with the correct compatibility between matrix and filler characteristics, can act as an inexpensive castable composite refractory [55].

## 3. Chloride Ion Penetrability and Corrosion Potential of Geopolymer Concrete

Rapid chloride ion permeability test (RCPT) is often used as a standard test to assess the chloride resistance of concrete in severe exposure conditions [56]. In an RCPT, the liquid that functions as an electrolyte, which flows through the pore spaces of concrete, is tested for its current rate. As the amount and continuity of pores existing in concrete samples affect the passage of ions and thus affect the current rate, it is expected that porous samples with continuous pores have high passing flow, and samples with low porosity have low passing flow [57]. It is notable that the low chloride ion penetrability of geopolymer concrete implies its low corrosion potential. The results of RCPT of the various types of geopolymer concrete were reported in several studies.

Ganesan et al. [58] compared the chloride resistance of various types of concrete, namely, geopolymer concrete (GPC), steel-fiber-reinforced geopolymer concrete (SFRGPC), conventional concrete (CC), and steel-fiber-reinforced concrete (SFRC) with reference to the ASTM C1202-19 [59] standard. The mix designs for the various types of concrete are specified in Table 2. The chloride ion penetrability values of all the concrete specimens under the study were assessed according to the total charge passed through them, and the results are shown in Table 3. It can be observed in Table 3 that the total charge-passed values for all the concrete specimens were within the range of 1000 to 2000 Coulombs, which indicates that they had low chloride ion penetrability. The CC and GPC had the highest and the lowest chloride ion penetrability values of 1764 and 1321 Coulombs, respectively. The findings were verified by their depths of chloride ion penetration and diffusion coefficients. As shown in Table 3, the CC and GPC had the highest and lowest respective values of depth of chloride penetration and diffusion coefficient. Addition of reinforcement with steel fibers in the geopolymer concrete resulted in an increase of its chloride ion penetrability. With the lower chloride ion penetrability value, it was confirmed that the GPC had better quality than the CC.

**Figure 7:**
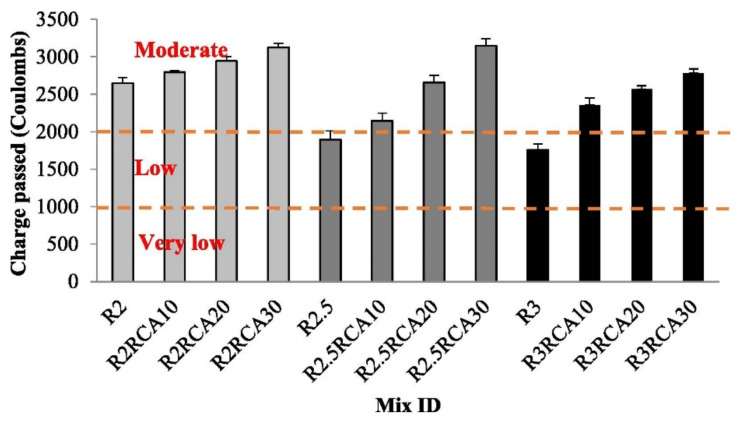
Effect of sodium-silicate-to-sodium-hydroxide ratio, normal-coarse aggregate, and recycled coarse aggregate (RCA) replacement on the chloride permeability of geopolymer concrete with reference to Table 4. Reproduced with permission from [60], [Construction and Building Materials]; published by [Elsevier], [2019].

The effect of sodium-silicate-to-sodium-hydroxide ratio and the type of coarse aggregate on the chloride ion penetrability of metakaolin-based geopolymer concrete was studied by Koushkbaghi et al. [60]. The two types of coarse aggregate that were incorporated in the geopolymer concrete are normal-coarse aggregate (NCA) and recycled coarse aggregate (RCA). Table 4 shows the mix proportions of the geopolymer concrete. The trend of chloride ion penetrability of the geopolymer concrete is depicted in Figure 7. It is shown in Figure 7 that the chloride ion penetrability increased with each percentage addition of RCA as a partial replacement of NCA in the geopolymer concrete. It is also evident in Figure 7 that all the geopolymer concrete specimens with RCA had total charge-passed-values between 2000 and 4000. Such results classified them as concrete with moderate chloride ion penetrability in accordance to the standard of ASTM C 1202-19 [59]. Koushkbaghi et al. [60] stipulated that the main reasons behind this result could be attributed to the high water absorption and porous structure of RCA. Another obvious observation from Figure 7 is that increasing the sodium-silicate-to-sodium-hydroxide ratio from 2 to 3 in the geopolymer concrete led to a decrease in the chloride ion penetrability. This behavior can be attributed to the penetration of sodium ions into the matrix, which increases alkalinity of the pore solution [60]. Further research needs to be done on the implication of aggregate size of geopolymer concrete on its chloride resistance, since good grading of the aggregate is known to minimize its porosity and chloride infiltration.

**Figure 8:**
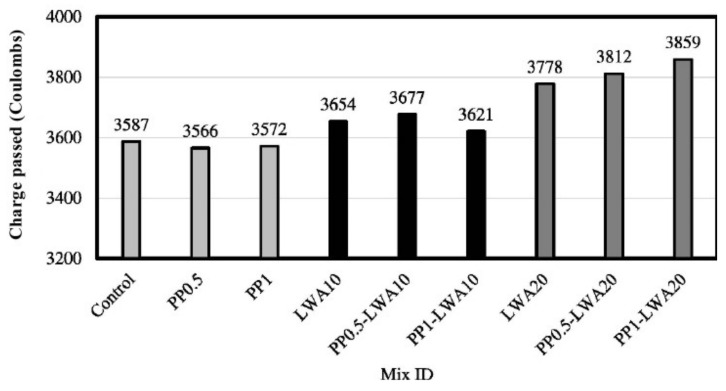
Rapid chloride ion penetrability test results of geopolymer concrete specimens with the variation of PP fiber and LWA percentages based on Table 5. Reproduced with permission from [61], [Construction and Building Materials]; published by [Elsevier], [2019].

The chloride resistance of fiber-reinforced lightweight geopolymer concrete specimens based on rice husk ash and nano-alumina was examined by Mohseni et al. [61]. Scoria particles and polypropylene fibers (PP) were utilized as the lightweight aggregate (LWA) and reinforcing material, respectively, in the geopolymer concrete specimens. The mixture proportions and RCPT results at 90-day curing of the geopolymer concrete specimens are shown in Table 5 and Figure 8, respectively. It is seen in Figure 8 that all the geopolymer concrete specimens had the total charge passed through them between 2000 to 4000 Coulombs. Therefore, the chloride ion penetrability of all the geopolymer concrete specimens can be rated as moderate according to the ASTM C 1202-19 [59] standard. Additionally, it is depicted in Figure 8 that addition of polypropylene fibers up to 1% in the geopolymer concrete specimen did not increase the total charge passed in the RCPT. The total charge-passed-values of the specimens for both control and geopolymer concrete improved with 1% polypropylene fibers (PP1) and were found to be 3587 and 3572 Coulombs, respectively. This showed that the polypropylene fibers were efficient at reducing the porosity of the geopolymer concrete, thereby enhancing its chloride resistance. On the other hand, the geopolymer concrete specimen improved with 1% polypropylene fibers and 20% partial replacement of coarse aggregate (CA) with lightweight aggregate (LWA), and had a total charge-passed-value of 3859 Coulombs. This value is three-fold higher than that of the control geopolymer concrete specimen. The finding indicated that the LWA is inferior at reducing the chloride permeability of the geopolymer concrete compared to CA.

**Figure 9:**
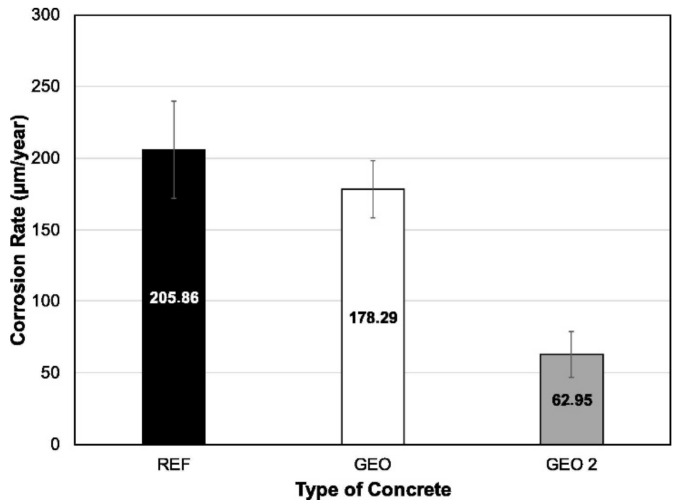
Corrosion rate of steel rebars inserted into the specimens of geopolymer and conventional concrete, measured after the corrosion potential test. (Note: The mixture proportions of the concrete specimens are based on Table 6). Reproduced with permission from [62], [Construction and Building Materials]; published by [Elsevier], [2021].

Amorim et al. [62] compared the corrosion rate of OPC concrete specimen with those of metakaolin based geopolymer concrete specimens. Their mixture proportions and average corrosion rates are presented in Table 6 and Figure 9, respectively. Each concrete specimen was reinforced with eight steel rebars. It was proven that the OPC concrete (REF) specimen had the highest average corrosion rate of 205.86 μm year^−1^. This was followed by the geopolymer concrete specimens of GEO and GEO2 with the average corrosion rates of 178.29 and 62.95 μm year^−1^, respectively. The decline in their corrosion rates may be associated with the greater capacity for fixing chlorides in this matrix [62]. The results proved that the geopolymer concrete provided better corrosion protection compared to the OPC concrete. The lowest average corrosion rate of GEO2 could be attributed to its water to sodium hydroxide ratio of 9.39, which is 2.77 lower than the molar ratio of GEO. The lowest molar ratio of GEO2 implied that it had a higher level of alkalinity and smaller void spaces that effectively reduced its corrosion potential due to the ingress of chemicals.

It can be epitomized from Section 3 that low porosity of geopolymer concrete reduced its chloride ion penetrability and corrosion potential. In this regard, the type of raw materials and the mixture proportions of geopolymer concrete were the contributing factors that influenced its degree of chloride ion penetrability. Robust aggregate, effective fiber reinforcement, and high level of alkalinity of admixtures could enhance the resistivity of geopolymer concrete against chloride attack, thereby reducing the corrosion tendency of its reinforcing steel bars. Geopolymer concrete was noticed to be less permeable to chloride intrusion than OPC concrete. This implied that it could offer better resistance to corrosion attack on its steel reinforcement compared to OPC concrete. This behavior may be associated with the greater fixation power of chlorides by the geopolymer matrix, which reduces the number of free chlorides available, thus reducing the flow of chloride percolation [62].

## 4. Acid Resistance of Geopolymer Concrete

For geopolymer concrete, the acid attack may be associated with the depolymerization of aluminosilicate network structure and the liberation of silicic acid (Si(OH)_4_) [63]. By comparison, OPC concrete is known to deteriorate after exposure to an acidic environment with a pH below 6.5. The degradation of OPC concrete by acid is caused by the dissolution of its cementation particles and calcium-rich aggregate. This could be triggered by the formation of water-soluble calcium compounds due to the leaching action on the OPC concrete by the acid. From durability perspective, it is important to review the acid resistance of geopolymer concrete in comparison with OPC concrete.

**Figure 10:**
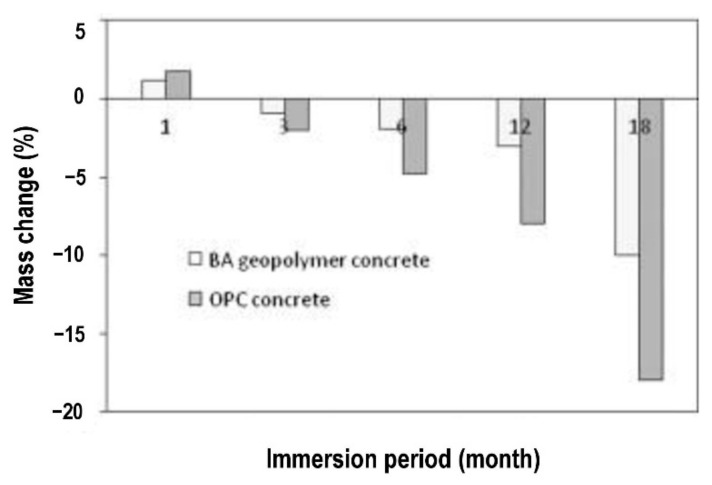
Mass change of concrete specimens in sulfuric acid for 18 months (note: BA, blended ash; OPC, Ordinary Portland Cement). Reproduced with permission from [64], [Construction and Building Materials]; published by [Elsevier], [2013].

**Figure 11:**
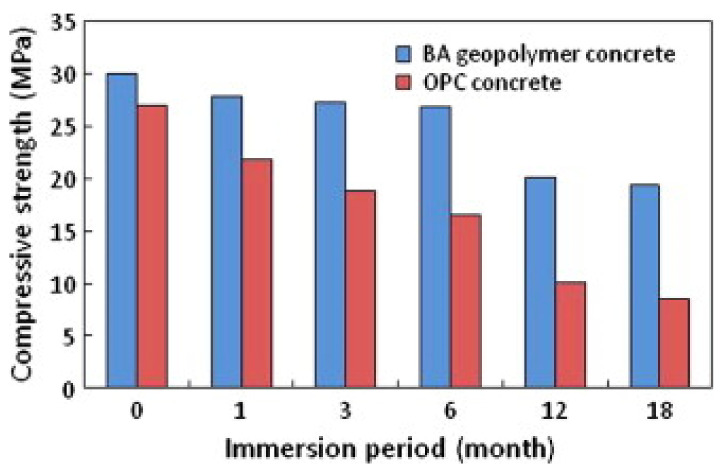
Compressive strength of concrete specimens exposed to sulfuric acid for 18 months. (Note: BA, blended ash; OPC, Ordinary Portland Cement). Reproduced with permission from [64], [Construction and Building Materials]; published by [Elsevier], [2013].

Ariffin et al. [64] evaluated the sulfuric acid resistance of blended ash geopolymer concrete. Pulverized fuel ash (PFA) and palm oil fuel ash (POFA) were mixed to form the blended ash (BA). Figure 10 and Figure 11 show the mass change and compressive strength of concrete specimens after exposure to 2% sulfuric acid up to 18 months. It is seen in Figure 10 that the mass of OPC and BA geopolymer concrete specimens increased by 1.8 and 1.2% respectively at 1 month exposure to the acid. After that, there was a consistent mass loss of each concrete specimen with each increase in the duration of acid exposure. By 18 months of exposure to the acid, the mass loss of the geopolymer concrete specimen was realized to be 8% less than that of the OPC concrete specimen. This proved that the acid degradation of the geopolymer concrete specimen was greater than that of the OPC concrete specimen. The impact of sulfuric acid attack on the mass loss of the concrete specimens could be verified by the consistent decrease in their compressive strength, as shown in Figure 11. The compressive strength loss of the geopolymer concrete specimen was noticed to be 35% at 18 months of sulfuric acid exposure. By comparison, during the same period of sulfuric acid exposure, the compressive strength loss of the OPC concrete specimen was realized to be 68%, which was much higher. This implied that the acid attack on the calcium silicate and calcium aluminate bonding in the OPC concrete happened at a faster rate than its destruction of aluminosilicate bonding in the geopolymer concrete. Such phenomenon weakened the concrete specimens due to their acid exposure over time, causing them to deform and crack.

**Figure 12:**
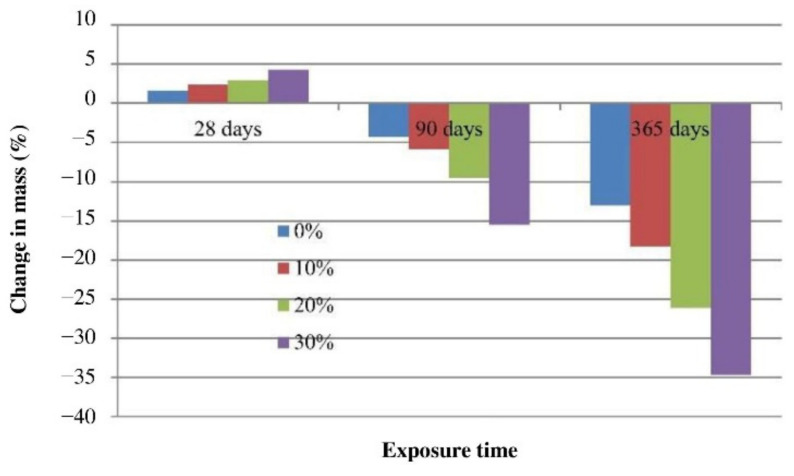
Mass loss of geopolymer concrete specimens exposed to sulfuric acid solution with 0, 10, 20, and 30% Ordinary Portland Cement (OPC). Reproduced with permission from [65], [Construction and Building Materials]; published by [Elsevier], [2017].

Mehta and Siddique [65] studied the effect of exposure to sulfuric acid on fly-ash-based geopolymer concrete. The mix designs of the geopolymer concrete specimens were developed by adding OPC as a calcium source and as a partial replacement of fly ash. The fly ash content was variably substituted with OPC at 0, 10, 20, and 30% in the geopolymer concrete specimens. The geopolymer concrete specimens were submerged in a 2% sulfuric acid solution with a pH of 1 for a duration up to 365 days. Their changes in mass over time were measured, and the results are depicted in Figure 12. At a 28-day exposure to sulfuric acid, the geopolymer concrete specimens with 0, 10, 20, and 30% OPC were found to have mass gain of 1.61, 2.34, 2.89, and 4.26%, respectively. This was attributed to the fact that the geopolymer concrete specimens were subjected to oven curing for 24 h at 80 °C, which created the extra pores in the concrete matrix [65]. The acid solution seeped through the pore spaces of the geopolymer concrete specimens, and this caused their weights to increase at the duration of acid exposure. The mass gain of geopolymer concrete specimens at the similar duration of acid exposure was also reported in the study of Charkhtab Moghaddam et al. [66]. A continuous loss in mass of the geopolymer concrete specimens was noticed between 28 and 365 days of the acid exposure. At 365-day of acid exposure, the mass loss values of the geopolymer concrete specimens with 0, 10, 20, and 30% OPC were observed to be 12.97, 18.26, 26.11, and 34.59%, respectively. It was clear from the study that the geopolymer concrete specimen without the OPC had the lowest rate of acid degradation due to the fact that the aluminosilicate compound had a better toughness against acid destruction compared to those of the calcium silicate hydrate and calcium aluminate hydrate compounds in the geopolymer concrete specimens with OPC. When the specimens were exposed to sulfuric acid solution and calcium hydration products, they are more likely to react with the acid and convert to calcium sulfate and calcium sulfo-aluminate [65]. This led to the progressive damage and cracking of the geopolymer concrete specimens during acid exposure.

**Figure 13:**
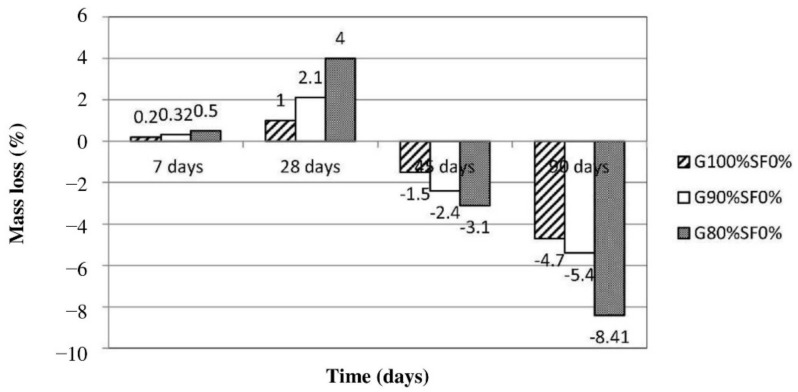
Mass loss of geopolymer concrete specimens exposed to sulfuric acid (note: G100%SF0%, mix proportions: 408 kg fly ash, 1347 kg coarse grains, 546.47 kg fine grains, 6.2 kg super lubricant, and 163.2 kg alkyl solution; G90%SF0%, mix proportions: 40.8 kg cement, 367.2 kg fly ash, 19.67 kg retail tires, 1347 kg coarse grains, 507.38 kg fine grains, 6.2 kg super lubricant, and 163.2 kg alkyl solution; G80%SF0%, mix proportions: 81.6 kg cement, 326.4 kg fly ash, 19.67 kg retail tires, 1347 kg coarse grains, 522.93 kg fine grains, 6.2 kg super lubricant, and 163.2 kg alkyl solution). Reproduced with permission from [66], [Construction and Building Materials]; published by [Elsevier], [2021].

Investigation into the sulfuric acid resistance of fly-ash-based geopolymer concrete was also carried out by Charkhtab Moghaddam et al. [66]. The mixture proportions of the geopolymer concrete specimens were designed with 0, 10, and 20% OPC as a partial fly ash replacement, and with crumb rubber equivalent to 10% volume of the fine grains. Dry curing method was applied to cure the geopolymer concrete specimens at a temperature of 60 °C for 28 days. Figure 13 shows the mass changes of the geopolymer concrete specimens after exposure to the acid attack (pH = 1) between 7 and 90 days. Logically, there was a progressive increase in the mass gain of the geopolymer concrete specimens between 7 and 28 days of the acid exposure. This happened due to the filling of the acid in the void spaces of the geopolymer concrete specimens. Between 28 and 90 days of the acid exposure, there was a consistent increase in the mass loss of the geopolymer concrete specimens. At 90-day acid exposure, the G100%SF0% specimen had the least mass loss of 4.7% compared to the mass losses of G90%SF0% and G80%SF0% specimens, which were 5.4 and 8.41%, respectively. The discovery is in agreement with that of Mehta and Siddique [65]—that the breakage of the aluminosilicate amorphous structure of geopolymer by the acid took place at a slower rate compared to the acid degradation of the cementation structure (calcium silicate hydrate and calcium aluminate hydrate) induced by OPC. This confirmed that the geopolymer concrete without OPC had better material stability against sulfuric acid aggression compared to the geopolymer concrete with OPC as a partial substitute of fly ash.

The main discoveries on geopolymer concrete’s resistance to sulfuric acid for three published works are summarized in Table 7. Table 8 shows the geopolymer concrete admixtures relevant to Table 7. Based on the forest plot of Table 9, which is related to Table 7 and Table 8, it was calculated that the average compressive strength reduction of the geopolymer concrete after the acid exposure is 14.91%. After submerging fly-ash-based geopolymer concrete with 2% sulfuric acid for 28 days, Abhilash et al. [67] found that its compressive strength was marginally reduced from 35.87 to 33.82 MPa (5.72%). In the same study, exposure of the geopolymer concrete to 2% hydrochloric acid for 28 days resulted in a small reduction of its compressive strength from 35.85 to 33.98 MPa (5.22%). It could be deduced from these literature findings that regardless of the acid type, there was not much compressive strength decrement of the geopolymer concrete due to the weak acid attack. Valencia-Saavedra et al. [68] examined the compressive strength of fly-ash-based geopolymer concrete with granulated blast furnace slag as an extra alkaline activator, after exposure to a sulfuric acid solution of 1 molar concentration for 28 days. From the initial strength value of 42.92 MPa, the compressive strength of the geopolymer concrete declined by 20% after the acid exposure. By comparison, the compressive strength of OPC concrete was drastically reduced by 55% from the initial value of 30.93 MPa after exposure to the same acid solution and duration. The study outcome revealed that the OPC concrete had a much lower sulfuric acid resistance compared to the geopolymer concrete. Due to acid attack, the calcium leaching from the OPC concrete caused a more severe degradation effect compared to the deterioration as a result of aluminosilicate depolymerization in the geopolymer concrete. In the study of Çevik et al. [69], it was affirmed that fly ash and nano-silica could be effectively combined as a precursor for producing acid-resistant geopolymer concrete. By exposing the fly ash and nano-silica based geopolymer concrete to 5% sulfuric acid for one month, its compressive strength decreased from 48.4 to 39.2 MPa (19% reduction). Without nano-silica, the compressive strength of the geopolymer concrete lessened from 51.63 to 35.3 MPa (32% reduction) after subjection to a similar acid condition. Such discovery provided evidence that nano-silica played an effective role in reducing the severity of geopolymer concrete deterioration as a result of the acid attack. This confirmed the efficient combination of nano-silica and fly ash as a precursor for the geopolymerization of the concrete that could significantly reduce the damage caused by the acid. Furthermore, the capability of the nano-silica to clog the pores of the geopolymer concrete helped to reduce the acid ingress, and this contributed to its lower compressive strength reduction after the acid exposure.

In summary of Section 4, geopolymer concrete has been proven to have better chemical stability than OPC concrete due to the fact that its geopolymerized bonding is more robust than the Portland cementation bonding to fend off the deterioration caused by sulfuric acid. Detailed research on the acid resistance of geopolymer concrete should be expanded to include other types of acid such as carbonic and hydrochloric acids. It must be noted that weak carbonic acid is formed as a result of carbon dioxide dissolved in rain water. Concrete is subjected to weathering process inclusive of exposure to rain water. Other than that, resistance to hydrochloric acid is a significant durability issue for concrete used in environments with caustic conditions [6]. The choice of acid solutions and their concentrations was based on practical utilization of concrete as construction material in sewage, pipes, mining, and food processing industries [70]. Geopolymer concrete manufactured by blending fly ash and kaolin show excellent durability properties with higher resistance to acid attack [71]. In this regard, it is interesting to explore the acid resistance of geopolymer concrete made of other aluminosilicate sources such as volcanic clay and wood ash.

## 5. Abrasion Resistance of Geopolymer Concrete

Abrasion wearing of concrete surface occurred due to mechanical scraping, wearing, or skidding of objects on the concrete surface, and it is one of the key considerations of concrete durability [72]. The compressive strength of geopolymer concrete, the toughness of aggregate, and the hardness of geopolymer binder are the contributing factors to the abrasion resistance of geopolymer concrete. With respect to that, the abrasion resistance of geopolymer concrete can be measured from its weight loss due to surface abrasion and its depth of wear.

**Figure 14:**
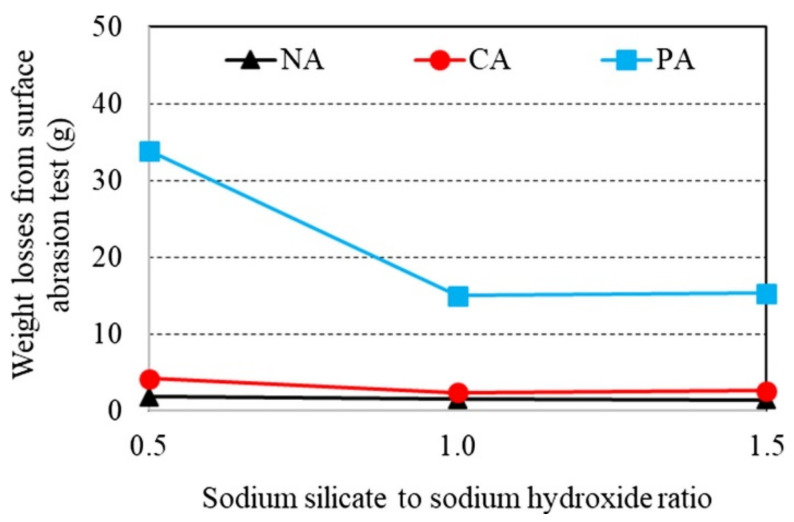
Weight losses from surface abrasion test and sodium-silicate-to-sodium-hydroxide ratios for various types of aggregates (note: NA, crushed natural limestone aggregate and river sand; CA, clay brick aggregate; and PA, pumice aggregate). Reproduced with permission from [73], [Construction and Building Materials]; published by [Elsevier], [2018].

Wongsa et al. [73] compared the effects of natural limestone and river sand aggregate (NA), clay brick aggregate (CA), and pumice aggregate (PA) on the abrasion resistance of lightweight geopolymer concrete. The geopolymer binder of the concrete was developed by mixing the aluminosilicate source in the form of high-calcium fly ash with the alkali activator in the form of sodium silicate and sodium hydroxide. Figure 14 shows the influence of the sodium-silicate-to-sodium-hydroxide ratios on the weight losses of the geopolymer concrete specimens from surface abrasion tests. The test results revealed that the weight loss of the geopolymer concrete specimen decreased with each increase of its sodium-silicate-to-sodium-hydroxide ratio. For instance, it has been proven from the tests that the weight loss of the geopolymer concrete specimen with CA reduced from 4.17 to 2.63 g as its sodium-silicate-to-sodium-hydroxide ratio increased from 0.5 to 1.5. At any sodium-silicate-to-sodium-hydroxide ratio, the geopolymer concrete specimen with NA was tested to have the lowest weight loss. This implied that the geopolymer concrete specimens with NA had the highest resistance, and this was followed by the geopolymer concrete specimens with CA and PA. It should be noted that the increases in the sodium-silicate-to-sodium-hydroxide ratio strengthened the geopolymer paste and bonding between aggregate and geopolymer paste [73]. This was assured by the fact that with an increase of sodium-silicate-to-sodium-hydroxide ratio from 0.5 to 1.5 in the geopolymer concrete specimens with CA, their compressive strength and splitting tensile strength increased from 8.2 to 18.3 MPa and from 0.6 to 1.6 MPa, respectively.

**Figure 15:**
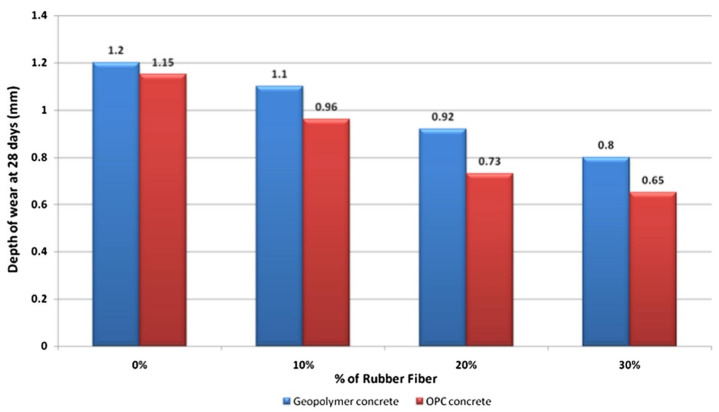
Depth of wear for OPC and geopolymer concrete specimens (Note: OPC, Ordinary Portland Cement). Reproduced with permission from [74]. [Construction and Building Materials]; published by [Elsevier], [2019].

In another study, Luhar et al. [74] compared the abrasion resistance of rubberized geopolymer concrete with that of OPC concrete in term of its depth of wear at 28 days age. Figure 15 indicates the relationship between the depth of wear of the concrete specimens at 28 days age and their percentages of rubber fibers based on the abrasion tests. It can be analyzed from the figure that the depth of wear reduced with each increase in the percentage of rubber fibers of the concrete specimen. Without rubber fiber, the depths of wear of the geopolymer and OPC concrete specimens were observed to be 1.2 and 1.15 mm, respectively. With 30% rubber fibers, the depths of wear of the geopolymer and OPC concrete specimens were tested to be 0.8 and 0.65 mm, respectively. The marginal difference in the depths of wear implied that the rubberized geopolymer concrete specimen had a compatible abrasion resistance with that of the OPC concrete specimen. The decrease in the depth of wear with each increase in the percentage of fibers for both types of concrete specimen showed that the rubber fibers’ addition enhanced their abrasion resistance.

Ramujee and Potharaju [75] explored the abrasion resistance of both geopolymer and OPC concretes, and the results were expressed in terms of average depth of wear, as shown in Table 10. The mix designs for both types of concrete are given in Table 11. The geopolymer concrete was evaluated to have average depth of wear of 2.8 and 4.4 mm at the respective abrasion test times of 12 and 24 h. In comparison, the OPC concrete was tested to have greater average depth of wear of 4.5 and 7.2 mm at the abrasion test times of 12 and 24 h, respectively. The literature results proved that the geopolymer concrete had a slower rate of surface degradation by scraping away due to abrasion compared to OPC concrete. The abrasive hardness of geopolymer concrete was also confirmed in the published work of Naveen Kumar and Ramujee [76].

**Figure 16:**
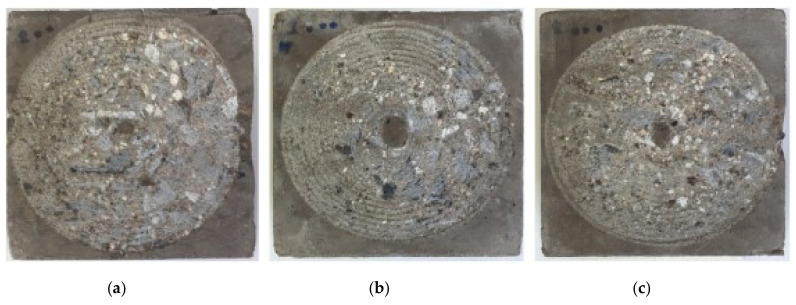
The surface conditions of high calcium fly ash geopolymer concrete at 0.75 liquid alkaline/ash ratio after surface abrasion test with (**a**) 0% recycled asphaltic concrete aggregate, (**b**) 20% recycled asphaltic concrete aggregate, and (**c**) 40% recycled asphaltic concrete aggregate. Reproduced with permission from [77], [Case Studies in Construction Materials]; published by [Elsevier], [2021].

Wongkvanklom et al. [77] evaluated the surface abrasion resistance of high calcium fly ash geopolymer concrete with varying percentages of recycled asphaltic concrete aggregate (RACA) and liquid alkaline/ash ratios. In the geopolymer concrete admixtures, RACA was applied to partially replace coarse limestone aggregate at 0, 20, and 40%, and the liquid alkaline/ash ratios were varied from 0.45 to 0.75. Table 12 indicates the surface abrasion weight loss of the geopolymer concrete. The relevant mix designs of the geopolymer concrete are shown in Table 13. In general, the surface abrasion weight loss of the geopolymer concrete decreased with each increase in the percentage of RACA. The geopolymer concrete with 0.55 liquid alkaline/ash ratio had the best surface abrasion result. For the geopolymer concrete with 0, 20, and 40% RACA at 0.55 liquid alkaline/ash ratio, it was found that the surface abrasion weight loss declined at 2.14, 2.11, and 1.88 g, respectively. The greatest improvement in the surface abrasion resistance showed that increasing recycled asphaltic concrete aggregate in the geopolymer concrete admixture was beneficial at toughening the surface of geopolymer concrete through the reduction of its pore spaces. The surface damage of the geopolymer concrete with the highest RACA content was effectively minimized after exposure to cutting with a rotating blade in the test. Such fact was validated by examining the photos of the geopolymer concrete after the surface abrasion tests (Figure 16). Without RACA, the geopolymer concrete showed the greatest surface damage (Figure 16a). Moderate surface damage could be seen in the geopol-ymer concrete with 20% RACA (Figure 16b). The geopolymer concrete with 40% RACA was observed to have the least surface damage (Figure 16c).

Based on the overview of Section 5, it can be summarized that strong and less porous aggregate could play a key role in developing high abrasion resistance of geopolymer concrete. In relation to that, it is appealing to explore the application of robust raw material such as basalt aggregate and recycled material such as recycled granite aggregate to enhance the abrasion resistance of geopolymer concrete. Due to the reinforcement effect, rubber fibers could be optimized with the binder in the geopolymer concrete to minimize its porosity and maximize its compressive strength so that it could be developed to resist abrasion. Other types of fibers such as steel and aluminum fibers could be researched as reinforcements of geopolymer concrete for the improvement of its abrasion resistance.

## 6. Morphological and Chemical Properties of Durable Geopolymer Concrete

Dense structure is responsible for the increased strength characteristics of the concrete due to geopolymerization [78]. In this section, the morphological properties of durable geopolymer concrete samples were visualized from a detailed review of their scanning electron micrographs. Their chemical properties after acid exposure were evaluated by reviewing their X-ray diffraction (XRD) test results. The review of the microstructures and chemical compounds of the geopolymer concrete samples could provide insight into their capability to resist thermal and chemical degradations.

**Figure 17:**
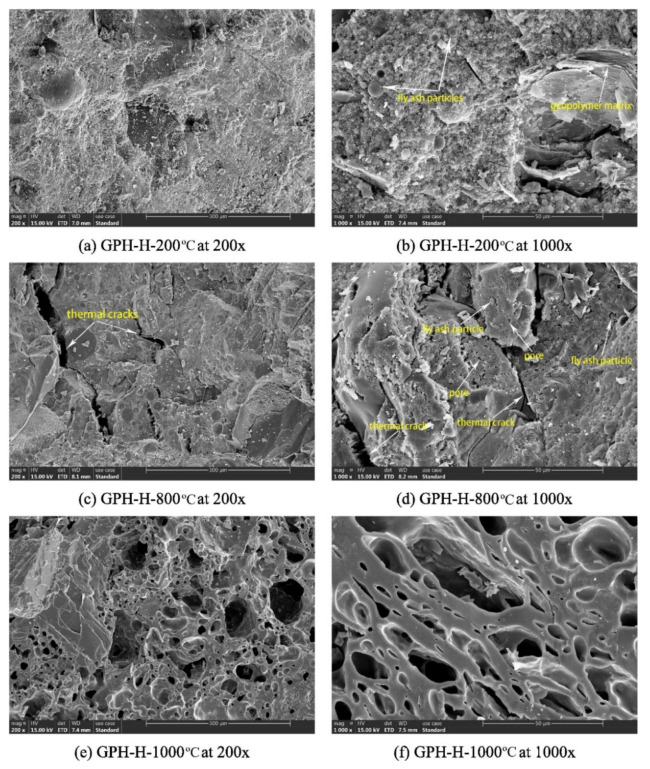
Scanning electron micrographs of GPH-H group geopolymer concrete samples at different elevated temperatures (Note: GPH-H, mix proportions: 1201 kg m^−3^ coarse crushed basalt aggregate, 539 kg m^−3^ natural siliceous sand, 460 kg m^−3^ fly ash, 150 kg m^−3^ sodium silicate, 50 kg m^−3^ sodium hydroxide, 3-sodium-silicate-to-sodium-hydroxide ratio, sodium hydroxide solution molarity of 14, 80 °C curing temperature, and 5.21% water content; 200×, 200× magnification; 1000×, 1000× magnification). Reproduced with permission from [22], [Construction and Building Materials]; published by [Elsevier], [2020].

**Figure 18:**
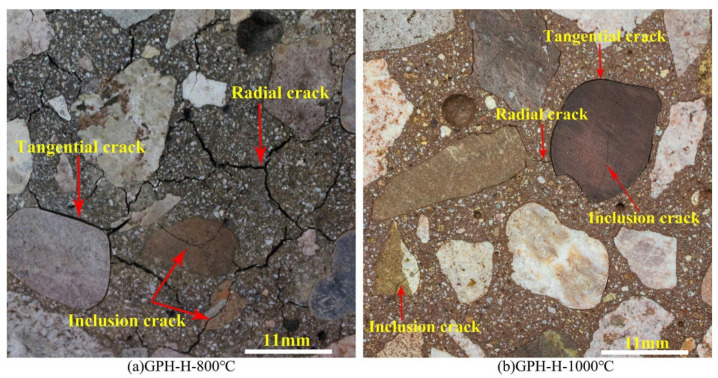
Thermal macro-cracks on part of the geopolymer concrete specimen cross-section at (**a**) 800 °C and (**b**) 1000 °C (Note: GPH-H, Mix proportions: 1201 kg m^−3^ coarse crushed basalt aggregate, 539 kg m^−3^ natural siliceous sand, 460 kg m^−3^ fly ash, 150 kg m^−3^ sodium silicate, 50 kg m^−3^ sodium hydroxide, 3-sodium-silicate-to-sodium-hydroxide ratio, sodium hydroxide solution molarity of 14, 80 °C curing temperature, and 5.21% water content; 200×, 200× magnification; 1000×, 1000× magnification). Reproduced with permission from [22], [Construction and Building Materials]; published by [Elsevier], [2020].

Zhang et al. [22] assessed the microstructures of fly-ash-based geopolymer concrete samples at elevated temperatures. Figure 17 shows the scanning electron micrographs of the geopolymer concrete samples exposed to 200, 800, and 1000 °C. At 200 °C, the microstructures of the geopolymer concrete sample are characterized by a dense and intact morphology with little pore spaces (Figure 17a,b). Similarly, dense and flat microstructures were observed in fly-ash-based geopolymer concrete researched by Zhao et al. [79] and Le et al. [80]. Zhao et al. [79] further discovered that that the dense microstructure of the geopolymer concrete remained intact after an exposure to 400 °C. After an exposure to 800 °C, however, some thermal micro-cracks could be seen in the microstructures of the geopolymer concrete samples (Figure 17c,d). A few unreacted fly ash particles could be observed in the micrographs as well. Additionally, loose microstructure was noticed on the surface of geopolymer concrete sample after it was subjected to a temperature of 800 °C based on the study of Zhao et al. [79]. The thermal micro-cracks were caused by extreme heat exposure, which reflected the serious deterioration of the geopolymer concrete specimen. At 1000 °C, no unreacted fly ash particle could be observed in the micrographs of the geopolymer concrete sample (Figure 17e,f). The big pores on the micrographs of the geopolymer concrete sample signified the destruction of its geopolymer matrix due to thermal stress. Figure 18a,b indicates the thermal macro-cracks on part of the geopolymer concrete specimen cross-section at elevated temperatures. Three types of thermal macro-crack were identified from the figure. They are radial, tangential, and inclusion cracks. According to Zhang et al. [22], the appearance of inclusion cracks indicated that the coarse aggregate of the geopolymer concrete specimen lost its ability to stand the thermal stress at 800 and 1000 °C. The thermal macro-cracks are more visible on the cross section of geopolymer concrete specimen exposed to 1000 °C compared to the one exposed to 800 °C, implying a more severe thermal degradation at the higher temperature. The porosity increased significantly after exposure to 400 °C and reached a maximum at 1000 °C, which could be directly attributed to increasing visible cracks [81].

**Figure 19:**
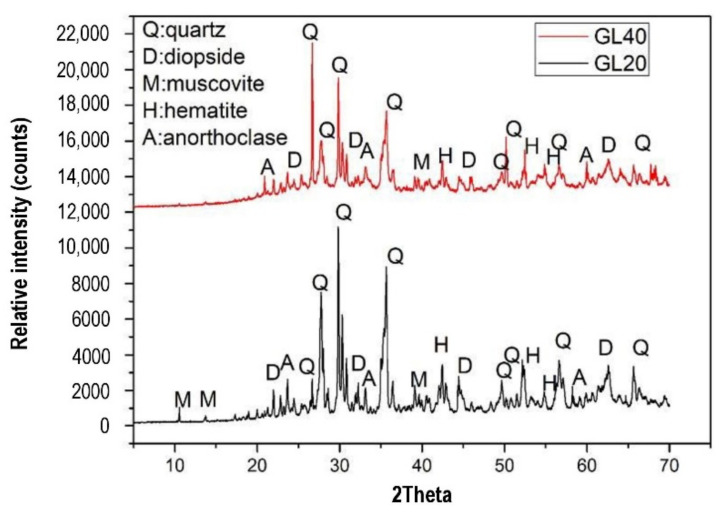
X-ray diffraction patterns of volcanic-ash-laterite-based geopolymer concrete samples cured at room temperature (28 °C). (Note: GL40, Mix proportions: 122.09 kg m^−3^ volcanic ash, 83.54 kg m^−3^ laterite, 357.15 kg m^−3^ river sand, 714.30 kg m^−3^ granite aggregate, and 0.80-sodium-silicate-to-sodium-hydroxide ratio; GL20, Mix proportions: 162.79 kg m^−3^ volcanic ash, 41.77 kg m^−3^ laterite, 357.15 kg m^−3^ river sand, 714.30 kg m^−3^ granite aggregate, and 0.80-sodium-silicate-to-sodium-hydroxide ratio). Reproduced with permission from [82], [Journal of Building Engineering]; published by [Elsevier], [2021].

**Figure 20:**
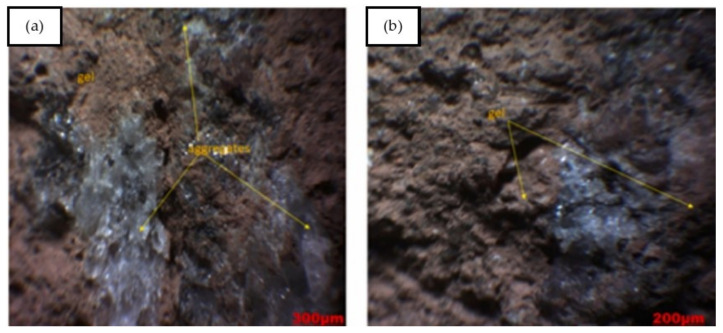
Optical observation of GL40 samples: (**a**) before and (**b**) after acid attack (note: GL40, mix proportions: 122.09 kg m^−3^ volcanic ash, 83.54 kg m^−3^ laterite, 357.15 kg m^−3^ river sand, 714.30 kg m^−3^ granite aggregate, and 0.80-sodium-silicate-to-sodium-hydroxide ratio). Reproduced with permission from [82], [Journal of Building Engineering]; published by [Elsevier], [2021].

Sontia Metekong et al. [82] performed X-ray diffraction analysis on volcanic ash and calcined-laterite-based geopolymer concrete samples. In the study, volcanic ash and calcined laterite were applied as the aluminosilicate sources of the geopolymer concrete. The results are shown in Figure 19. GL20 and GL40 are the two types of geopolymer concrete sample that were evaluated for their X-ray diffraction patterns. Their mixtures’ proportions are specified in the description of Figure 19. The mix designs of GL20 and GL40 were developed to have 20 and 40% calcined laterite by weight percentage of the aluminosilicate sources in the geopolymer concrete specimens respectively. In both GL20 and GL40 samples, the minerals of the volcanic ash inclusive of quartz, hematite, and anorthoclase could be traced from their high intensity peaks of X-ray diffraction. This could be justified by the fact that these mineral phases have been unaltered or partially dissolved in alkaline medium [82]. The unreacted minerals could function as filling agents that reinforced the geopolymer concrete, and this contributed to the concrete robustness. The geopolymer concrete samples were exposed to sulfuric acid at a pH of 1 for 19 weeks. The optical images of GL40 samples before and after the acid attack are shown in Figure 20. The microscopic image of Figure 20a shows that the particles of the geopolymer concrete were closely packed to each other to form intact and dense surface. Sontia Metekong et al. [82] further clarified that this densification could be due to the fact that the introduction of calcined laterite as additive in volcanic-ash-based geopolymer concrete extensively increased the reactive or amorphous phase, which allowed high polycondensation/polymerization between Si- and Al- oligomers, resulting in high Na-A-S-H geopolymer binder, contributing to good cohesion between aggregates and other particles. However, after the GL40 sample was exposed to the acid, pores were observed on the surface of the sample as indicated in the microscopic image of Figure 20b. The presence of these pores can be explained by the rupturing of polysialate (Si–O–Al) bonds by chemical attack of the matrix [82]. This caused leaching to take place, which gradually destroyed the geoploymer matrix and loosened the bonding among the geopolymer particles and aggregate, thereby reducing the strength of the geopolymer concrete.

**Figure 21:**
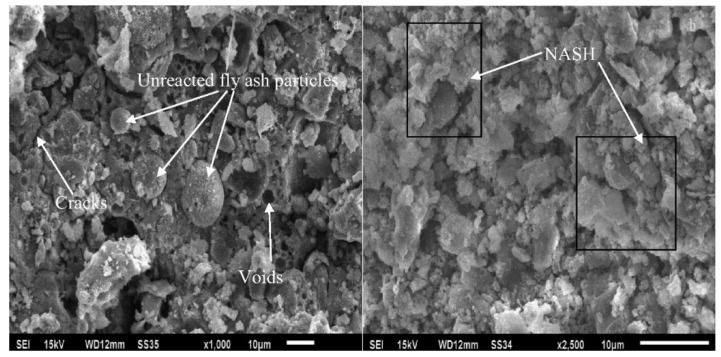
Scanning electron micrographs (SEM) of geopolymer concrete samples: (**a**) unexposed and (**b**) exposed to sulfuric acid solution for 365 days (note: mix proportions: 310 kg fly ash, 1204 kg coarse aggregate (crushed stone), 649 kg fine aggregate (river sand), 171 kg alkali solution (2.5-sodium-silicate-to-sodium-hydroxide ratio), and 6.2 kg naphthalene-based superplasticizer). Reproduced with permission from [65], [Construction and Building Materials]; published by [Elsevier], [2017].

The long-term effect of sulfuric acid attack on the morphology and X-ray diffraction pattern of fly-ash-based geopolymer concrete was reported by Mehta and Siddique [65]. Figure 21 reveals the scanning electron micrographs (SEM) of geopolymer concrete samples unexposed and exposed to sulfuric acid solution for 365 days. It can be observed from the micrograph of Figure 21a that the surface of the geopolymer concrete sample without the acid exposure was characterized by tightly packed particles with some voids and unreacted fly ash. The unreacted fly ash particles served as fillers of the geopolymer concrete that ensured its low porosity. Compared with the geopolymer concrete sample that was not exposed to acid, the geopolymer concrete sample exposed to acid had a lower particle packing efficiency (Figure 21b). However, in the geopolymer concrete sample exposed to acid, the main geopolymer binder remained intact. The geopolymer binder was the strength-contributing product, with strong alumina and silica bonds that were less affected by acid exposure [65].

**Figure 22:**
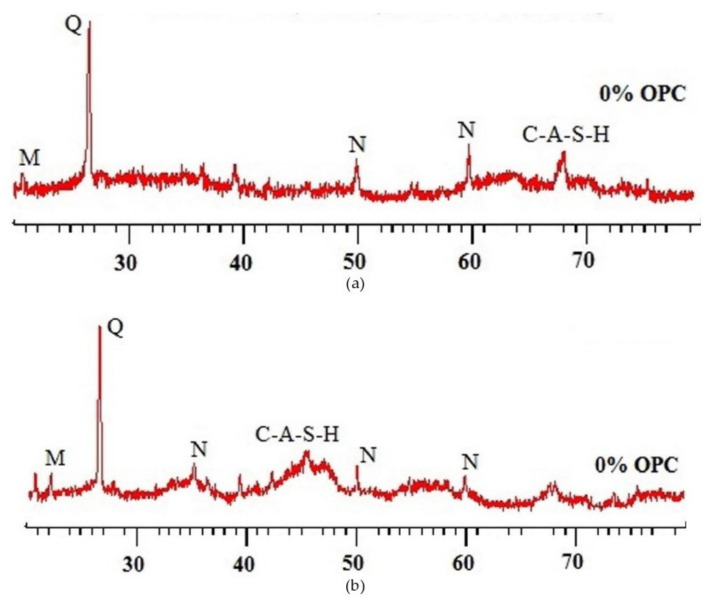
X-ray diffraction (XRD) patterns of geopolymer concrete samples: (**a**) unexposed and (**b**) exposed to sulfuric acid solution for 365 days (note: mix proportions: 310 kg fly ash, 1204 kg coarse aggregate (crushed stone), 649 kg fine aggregate (river sand), 171 kg alkali solution (2.5-sodium-silicate-to-sodium-hydroxide ratio), and 6.2 kg naphthalene-based superplasticizer). Reproduced with permission from [65], [Construction and Building Materials]; published by [Elsevier], [2017].

The chemical proofs of the fly-ash-based geopolymer concrete samples before and after sulfuric acid exposure could be traced from their X-ray diffraction patterns as illustrated in Figure 22. Without the acid exposure, there were significant peaks of X-ray diffraction of quartz (Q), mullite (M), nepheline (N), and calcium aluminosilicate hydrate (C-A-S-H) (Figure 22a). The presence of quartz in the sample showed the evidence of the aggregate in the geopolymer concrete. The existence of mullite (M), nepheline (N), and calcium aluminosilicate hydrate (C-A-S-H) in the sample revealed the proof of geopolymer binder with strong aluminosilicate bonds, which were responsible for the cementation of the geopolymer concrete. However, after the geopolymer concrete sample was exposed to the acid for 365 days, these minerals could still be observed in the sample but with lower X-ray diffraction intensities (Figure 22b). Such evidence pointed out that some degradation happened in the geopolymer concrete due to its geopolymer depolymerization as a result of the acid attack. Despite that, it was shown by Thokchom [83] that geopolymer concrete is better than OPC concrete to resist the degrading action caused by acid.

In Section 6, it can be summarized that in terms of durability, geopolymer concrete demonstrated more robust morphology and chemical composition compared to OPC concrete. This is explainable by the fact the sodium aluminosilicate bonds in geopolymer concrete are strong and therefore, putting up high resistance against both thermal and acid deteriorations. On the other hand, the calcium hydroxide of the OPC concrete is reactive to acid, and this caused leaching to occur. The leaching process could weaken the cementation bonds of the OPC concrete, resulting in the decrease of its strength.

## 7. Research Gap of Geopolymer Concrete

Critical reviews on geopolymer concrete were published in several articles. Assi et al. [84] provided an overview of the supply, demand, and cost of source materials of geopolymer concrete, including fly ash, slag cement, metakaolin, sodium hydroxide, sodium silicate, and silica fume. In the review, fly ash-based geopolymer concrete was justified as the most applicable one due to its economic and availability factors. In another review, Ng et al. [85] examined the microstructures and compressive strength development of geopolymer concrete. The review was limited to the analyses of the morphology, chemical, and compression properties of geopolymer concrete with various mixture proportions of different precursors. Jindal and Sharma [86] did a state-of-the-art review regarding the implication of nanomaterials on the properties of geopolymer concrete derived from industrial by-products. Although this review provided a comprehensive insight into the durability of geopolymer concrete improved with various types of nanomaterial inclusive of nano-silica, nano-alumina, nano titanium oxide, carbon nanotubes, and nanoclay, the utilization of nanomaterials for geopolymerization is not sustainable as their cost and availability override their benefits. In this review article, the endurance of geopolymer concrete with various mix designs and sources of raw materials was characterized in terms of compressive strength at an elevated temperature, chloride ion penetrability and corrosion potential, acid resistance, and abrasion hardness. Based on the evaluation of these reviews, there emerges a research gap between the geopolymerizing mechanism and the unexplored source of material that can enhance the durability of geopolymer concrete. In this regard, it is thought-provoking to research the durability performance of geopolymer concrete with heavy metal contaminated clay as an unexplored precursor. If proven true that the geopolymer concrete can encapsulate heavy metals from the contaminated clay in its bonding without leaching failure, it will pave a way for a green solution to the pollution of clay.

## 8. Conclusions and Future Recommendations

A comprehensive understanding on the durability behavior of geopolymer concrete could be achieved from this review. It can be concluded from this review that geopolymer concrete is durable and resistant to heat, chloride penetration, acid attack, and abrasion. An optimal addition of fibers and ultra-fine silica material such as nano-silica to geopolymer concrete could enhance its residual compressive strength. This could be achieved through the actions of reinforcing fibers and pore blocking of extremely-small-sized silica particles that prevented crack propagation in geopolymer concrete. In that regard, it is interesting for future research to explore the role of thermal insulating raw material such as fiberglass at enhancing the residual compressive strength of geopolymer concrete. A high degree of alkalinity in admixture; tough aggregate; and the optimal utilization of fibers and additives were the identified elements that could minimize the porosity and maximize the binding effect of geopolymer concrete, thereby protecting it from damage due to harsh environmental exposure. Additionally, it can be concluded from the review of the micrographs and X-ray diffraction analyses that geopolymer concrete deteriorated with time after exposure to an elevated temperature and a strong acid solution. However, its geopolymer binder was realized to be more stable than the cementation binder of OPC concrete to fend off heat and acid aggressions. As a recommendation, further research has to be carried out on the durability of geopolymer concrete, utilizing solid alkali activators such pentahydrate and anhydrous sodium metasilicate. The use of solid alkali activators in geopolymer mixes is less hazardous than the commonly used liquid activators and yields a “cement like” mix, where only water is added for its activation and hydration [87]. More investigation is needed to assess the long-term strength development of geopolymer concrete improved with various types of fiber and pozzolanic additive. This will provide a reliable correlation between the long-term strength gain of geopolymer concrete and its impact on the alkaline activation of aluminosilicate. For robustness compatibility assessment, the long-term strength gain of geopolymer concrete can be compared to that of OPC concrete with pozzolanic additive. It is also suggested that the research on geopolymer concrete to be expanded to include its structural integrity for field application. The geopolymer structural capacity to transmit loads from the roof to the foundation can then be compared to that of the structure made of OPC concrete.

## Figures and Tables

**Table 1:** Methods of geopolymerization and chemical compositions of geopolymer specimen admixture related to Figure 6.

Published Work	Method of Geopolymerization	Chemical Composition of Geopolymer Specimen Admixture
Rahmadina and Ekaputri [51]	Alkaline activation by sodium hydroxide and sodium silicate solutions with Class F fly ash as a precursor.	8 molar concentration of sodium hydroxide, 18.5% sodium oxide, 36.4% silica, and 45.1% water.
Jiang et al. [52]	Alkaline activation by sodium hydroxide and sodium silicate solutions with Class F fly ash as a precursor.	10 molar concentration of sodium hydroxide, 8.3% sodium oxide, 28.7% silica, and 63% water.
Payakaniti et al. [53]	Alkaline activation by sodium hydroxide and sodium silicate solutions with high calcium (Class C) lignite fly ash as a precursor.	10 molar concentration of sodium hydroxide, 12.53% sodium silicate by weight of sodium oxide, 30.24% silica, and 57.23% water.

**Table 2:** Mixture proportions for the preparation of concrete based on the work of Ganesan et al. [58] (note: GPC, geopolymer concrete; SFRGPC, steel-fiber-reinforced geopolymer concrete; CC, conventional concrete; SFRC, steel-fiber-reinforced concrete; CA, coarse aggregate; FA, fine aggregate; SP, naphthalene-based superplasticizer). Reproduced with permission from [58], [Construction and Building Materials]; published by [Elsevier], [2015].

Mix	Steel Fiber (%)	Fly Ash (kg m^−3^)	Sodium Silicate Solution (kg m^−3^)	Sodium Hydroxide Solution (kg m^−3^)	CA (kg m^−3^)	FA (kg m^−3^)	Water (kg m^−3^)	SP (kg m^−3^)	Cement (kg m^−3^)
GPC	0	408	103	41	1248	600	14.5	10.2	0
SFRGPC1	0.25	408	103	41	1248	600	16	10.2	0
SFRGPC2	0.5	408	103	41	1248	600	16	14.5	0
SFRGPC3	0.75	408	103	41	1248	600	18	14.5	0
SFRGPC4	1	408	103	41	1248	600	18	16	0
CC	0	0	0	0	1266	598	192	0	360
SFRC2	0.5	0	0	0	1266	598	192	4	360

**Table 3:** Results of bulk diffusion test and rapid chloride permeability test of concrete based on the work of Ganesan et al. [58] (note: GPC, geopolymer concrete; SFRGPC, steel-fiber-reinforced geopolymer concrete; CC, conventional concrete; SFRC, steel-fiber-reinforced concrete). Reproduced with permission from [58], [Construction and Building Materials]; published by [Elsevier], [2015].

Mix	Depth of Chloride Penetration (cm)	Diffusion Coefficient (m^2^ s^−1^)	Charge Passed (Coulombs)	Chloride Ion Penetrability as per ASTM
GPC	2.45	1.24 × 10^−11^	1321	Low
SFRGPC1	2.42	1.21 × 10^−11^	1445	Low
SFRGPC2	2.39	1.18 × 10^−11^	1392	Low
SFRGPC3	2.36	1.15 × 10^−11^	1566	Low
SFRGPC4	2.22	1.02 × 10^−11^	1762	Low
CC	2.49	1.28 × 10^−11^	1764	Low
SFRC2	2.47	1.26 × 10^−11^	1423	Low

**Table 4:** Mix proportions of geopolymer concrete (kg m^−3^) based on the work of Koushkbaghi et al. [60] (note: SS, sodium silicate; SH, sodium hydroxide; NCA, normal-coarse aggregate; and RCA, recycled coarse aggregate). Reproduced with permission from [60], [Construction and Building Materials]; published by [Elsevier], [2019].

Mix ID	Metakaolin	SS	SH	Aggregates		
				Fine Aggregate	NCA	RCA
R2 ^a^	400	90	45	600	1250	0
R2RCA10	400	90	45	600	1025	115
R2RCA20	400	90	45	600	910	230
R2RCA30	400	90	45	600	800	340
R2.5	400	115	45	600	1250	0
R2.5RCA10	400	115	45	600	1025	115
R2.5RCA20	400	115	45	600	910	230
R2.5RCA30	400	115	45	600	800	340
R3	400	135	45	600	1250	0
R3RCA10	400	135	45	600	1025	115
R3RCA20	400	135	45	600	910	230
R3RCA30	400	135	45	600	800	340

^a^ Numbers after R is SS/SH ratio and after RCA is recycled aggregate percentage.

**Table 5:** Mixture proportions for the preparation of geopolymer concrete specimens based on the work of Mohseni et al. [61] (note: RHA, rice husk ash; NA, nano-alumina; PP, polypropylene fibers; SS, sodium silicate; SH, sodium hydroxide; CA, coarse aggregate; LWA, lightweight aggregate). Reproduced with permission from [61], [Construction and Building Materials]; published by [Elsevier], [2019].

Mix ID	RHA (kg m^−3^)	NA (kg m^−3^)	PP (kg m^−3^)	SS (kg m^−3^)	SH (kg m^−3^)	Sand (kg m^−3^)	CA (kg m^−3^)	LWA (kg m^−3^)
Control	315	40	0	115	45	600	1250	0
PP0.5	315	40	2	115	45	600	1250	0
PP1	315	40	4	115	45	600	1250	0
LWA10	315	40	0	115	45	600	1125	55
PP0.5-LWA10	315	40	2	115	45	600	1125	55
PP1-LWA10	315	40	4	115	45	600	1125	55
LWA20	315	40	0	115	45	600	1000	110
PP0.5-LWA20	315	40	2	115	45	600	1000	110
PP1-LWA20	315	40	4	115	45	600	1000	110

**Table 6:** Mixture proportions (by weight) of the concrete specimens based on the work of Amorim et al. [62]. Reproduced with permission from [62], [Construction and Building Materials]; published by [Elsevier], [2021].

Mix	Material Consumption (kg m^−3^ of Concrete)						
	Portland Cement	Metakaolin	Sand	Gravel	Water	Sodium Hydroxide	Sodium Silicate
REF	402.69	0.00	736.92	954.37	241.60	0.00	0.00
GEO	0.00	340.55	623.21	807.10	0.00	170.27	323.52
GEO2	0.00	355.93	651.35	843.55	0.00	177.96	265.88

**Table 7:** Main discoveries on geopolymer concrete’s resistance to sulfuric acid.

Published Work	Key Discovery on Geopolymer Concrete’s Resistance to Sulfuric Acid
Abhilash et al. [67]	After submersion in a 2% sulfuric acid solution for 28 days, the geopolymer concrete was tested to have a 33.82 MPa compressive strength, which is 5.72% less than that without the acid exposure.
Valencia-Saavedra et al. [68]	After 28-day submersion in a sulfuric acid solution of 1 molar concentration, the geopolymer concrete was tested to have a 34.34 MPa compressive strength, which is 20% less than that without the acid exposure.
Çevik et al. [69]	After immersion in a 5% sulfuric acid solution for one month, the geopolymer concrete was tested to have a 39.2 MPa compressive strength, which is 19% less than that without the acid exposure.

**Table 8:** Geopolymer concrete admixtures relevant to Table 7.

Published Work	Alkaline Activator	Precursor	Geopolymer Concrete Admixture
Abhilash et al. [67]	Sodium silicate and sodium hydroxide	Fly ash	409 kg m^−3^ fly ash, 41 kg m^−3^ sodium hydroxide, 102 kg m^−3^ sodium silicate, 549 kg m^−3^ slag (fine aggregate), 903 kg m^−3^ crushed stone (coarse aggregate), and 387 kg m^−3^ coal washery rejects (coarse aggregate).
Valencia-Saavedra et al. [68]	Sodium silicate, sodium hydroxide, and granulated blast furnace slag	Fly ash	320 kg m^−3^ fly ash, 80 kg m^−3^ granulated blast furnace slag, 28.55 kg m^−3^ sodium hydroxide, 158.37 kg m^−3^ sodium silicate, 972.7 kg m^−3^ sand, and 704.4 kg m^−3^ gravel (Liquid/solid ratio = 0.48).
Çevik et al. [69]	Sodium silicate and sodium hydroxide	Fly ash and nano-silica	500 kg m^−3^ fly ash, 15 kg m^−3^ nano-silica, 225 kg m^−3^ sodium hydroxide and sodium silicate, 1150 kg m^−3^ coarse aggregate, 575 kg m^−3^ fine aggregate, and 6 kgm^−3^ superplasticizer.

**Table 9:** Forest plot of the main discoveries on geopolymer concrete’s resistance to sulfuric acid, with reference to Table 7 and Table 8.

**Published Work**	**Compressive Strength of Geopolymer Concrete without Acid Exposure (MPa)**	**Compressive Strength of Geopolymer Concrete with Acid Exposure (MPa)**	**Compressive Strength Reduction (%)**	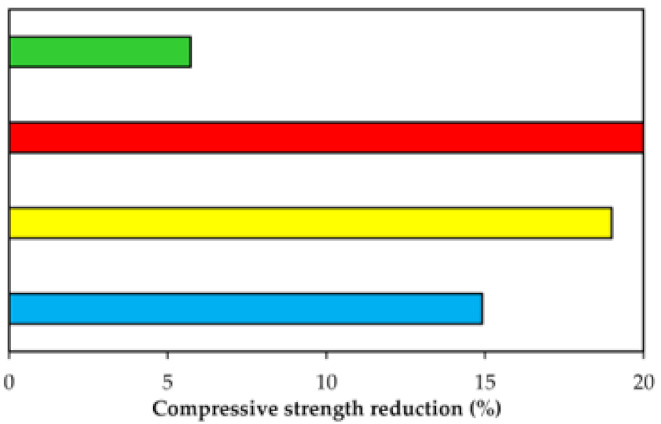
Abhilash et al. [67]	35.87	33.82	5.72	
Valencia-Saavedra et al. [68]	42.92	34.34	20	
Çevik et al. [69]	48.4	39.2	19	
**Average**			**14.91**	

**Table 10:** Summarized test results of concrete abrasion resistance from the study of Ramujee and Potharaju [75].

Time (Hours)	Average Depth of Wear (mm)	
	Geopolymer Concrete	OPC Concrete
12	2.8	4.5
24	4.4	7.2

**Table 11:** Concrete admixtures from the study of Ramujee and Potharaju [75] relevant to Table 9.

Concrete Type	Concrete Admixture
Geopolymer concrete	327 kg m^−3^ fly ash, 54.33 kg m^−3^ sodium hydroxide (8 M), 108.67 kg m^−3^ sodium silicate, 672 kg m^−3^ fine aggregate, 1248 kg m^−3^ coarse aggregate, and 22 kg m^−3^ water (Liquid/binder ratio = 0.50).
OPC concrete	327 kg m^−3^ fly ash, 328 kg m^−3^ ordinary Portland cement, 2 kg m^−3^ sulphonated napthalene based superplasticizer (GLENIUM B233), 672 kg m^−3^ fine aggregate, 1248 kg m^−3^ coarse aggregate, and 163 kg m^−3^ water (Liquid/binder ratio = 0.50).

**Table 12:** Weight losses by surface abrasion of high calcium fly ash geopolymer concrete from the published work of Wongkvanklom et al. [77]. Reproduced with permission from [77], [Case Studies in Construction Materials]; published by [Elsevier], [2021].

Liquid Alkaline/Ash Ratio	Surface Abrasion Weight Loss (g)		
	00RACA	20RACA	40RACA
0.45	2.08	2.22	2.11
0.55	2.14	2.11	1.88
0.65	3.16	2.27	2.06
0.75	4.16	3.21	3.16

**Table 13:** High calcium fly ash geopolymer concrete admixtures from the published work of Wongkvanklom et al. [77] relevant to Table 11. Reproduced with permission from [77], [Case Studies in Construction Materials]; published by [Elsevier], [2021].

Mix ID	Coarse Aggregate (kg m^−3^)		Sand (kg m^−3^)	Fly Ash (kg m^−3^)	Sodium Hydroxide (kg m^−3^)	Sodium Silicate (kg m^−3^)
	RACA	NA				
0.45LA—00RACA	0	1090	590	428	96.3	96.3
0.45LA—20RACA	220	875	590	428	96.3	96.3
0.45LA—40RACA	440	655	590	428	96.3	96.3
0.55LA—00RACA	0	1090	590	428	117.7	117.7
0.55LA—20RACA	220	875	590	428	117.7	117.7
0.55LA—40RACA	440	655	590	428	117.7	117.7
0.65LA—00RACA	0	1090	590	428	139.1	139.1
0.65LA—20RACA	220	875	590	428	139.1	139.1
0.65LA—40RACA	440	655	590	428	139.1	139.1
0.75LA—00RACA	0	1090	590	428	160.5	160.5
0.75LA—20RACA	220	875	590	428	160.5	160.5
0.75LA—40RACA	440	655	590	428	160.5	160.5

(Note: LA = liquid alkaline/ash ratio, RACA = recycled asphaltic concrete aggregate, NA = natural coarse limestone aggregate).

## Data Availability

No new data were created or analyzed in this study. Data sharing is not applicable to this article.
